# HKDC1 Silencing Inhibits Proliferation and Glycolysis of Gastric Cancer Cells

**DOI:** 10.1155/2023/3876342

**Published:** 2023-04-28

**Authors:** Chen Yu, Ting-ting Bao, Li Jin, Jian-wei Lu, Ji-feng Feng

**Affiliations:** ^1^Department of Integrated TCM & Western Medicine, Jiangsu Cancer Hospital & Jiangsu Institute of Cancer Research & the Affiliated Cancer Hospital of Nanjing Medical University, 42 Baiziting Road, Nanjing 210009, China; ^2^State Key Laboratory of Quality Research in Chinese Medicine & School of Pharmacy, Macau University of Science and Technology, Taipa, China; ^3^Department of Radiotherapy, Sichuan Cancer Hospital & Institute, Sichuan Cancer Center, School of Medicine, University of Electronic Science and Technology of China, 55 Renmin South Road, 610041 Chengdu, Sichuan, China; ^4^Department of Medical Oncology, Jiangsu Cancer Hospital & Jiangsu Institute of Cancer Research & The Affiliated Cancer Hospital of Nanjing Medical University, 42 Baiziting Road, Nanjing 210009, China

## Abstract

Gastric cancer (GC) is the third most lethal and fifth most common cancer in the world. In a variety of cancers, the hexokinase domain component 1 (HKDC1) is carcinogenic. This study was to investigate into how HKDC1 contributes to the development and progression of GC. Three different datasets (GSE103236, GSE13861, and GSE55696) were extracted from the Gene Expression Omnibus (GEO) database and then analyzed using the sva package. The R software was used to identify 411 differentially expressed genes (DEGs) in the pooled dataset. We discovered 326 glycolysis-related genes (glyGenes) in the cancer genome atlas-stomach adenocarcinoma (TCGA-STAD) cohort using gene set enrichment analysis set (GSEA). HKDC1 is one of the most prevalent glyGenes in GC tumor tissues and cells, as seen in the Venn diagram. According to the results of the Cell Count Kit-8 assay, the proliferation of AGS and MKN-45 cells decreased when HKDC1 was knocked down. Lack of HKDC1 in cells enhanced oxygen consumption and decreased glycolytic protein expression while suppressing glucose absorption, lactate production, ATP level, and extracellular acidification ratio. As an oncogene in gastric cancer development, HKDC1 influences cell proliferation and glycolysis.

## 1. Introduction

As one of the most common types of cancer, gastric cancer has the fifth-highest incidence rate (5.7%) [1]. Both the incidence and mortality rate of gastric cancer are the highest in China [2, 3]. The overall survival rate for gastric cancer is still around 30 percent, despite the finding that smoking and alcohol consumption are the principal drivers of gastric cancer. In contrast to the respiratory metabolism of the vast majority of normal cells, cancer cells prefer glycolysis as the source of energy even when nutrients are present [4]. This is a phenomenon known as the Warburg effect, and it is characterized by an increase in glucose uptake and lactate generation. The intermediate products produced during glycolysis not only offer nutrition for the expansion of tumor cells but also shield tumor cells, which increases the rate of malignant tumor cell proliferation and spread [5]. According to several studies, glycolysis results in the production of a variety of metabolites, including pyruvate, lactate, and ketone bodies, which are found in increased concentrations in the tissues and cells of gastric cancer [6–8]. Lowering glucose consumption while maintaining normal energy requirements significantly suppresses the Warburg effect on tumor cells [9], which slows down the growth of tumors.

The aerobic glycolysis and glycolysis pathways are both controlled by more than ten genes that encode key glycolysis enzymes and glucose transporters [10, 11]. As a consequence of this, transcription factors including HIF1, c-Myc, NF-B, and SIX1 that can directly influence the gene expression and protein activity of glucose transporters and key glycolysis enzymes play a significant role in the regulation of malignant tumor metabolism. In this study, the use of bioinformatics allowed for the identification of hexokinase domain component 1 (HKDC1) as a gene linked to gastric cancer, which was found to be closely associated with glycolysis. HKDC1 was found to potentially act as a fifth hexokinase and plays an essential role in preserving glucose homeostasis throughout the body [12]. On the other hand, abnormal expression of HKDC1 has been linked to the development of a wide variety of diseases and cancers. An example of this would be the overexpression of the gene HKDC1, which has been associated with metabolic inefficiency in hepatocytes, which in turn has been linked to nonalcoholic fatty liver disease [13]. In addition, there is accumulating evidence that HKDC1 may play an oncogenic function in cancers such as lymphoma, liver, breast, and colorectal cancers [14]. However, very little is known about the role that HKDC1 plays in the development of gastric cancer or the metabolic processes of tumors.

## 2. Materials and Methods

### 2.1. Gene Expression Microarray Datasets from the GEO Database and TCGA Database

For the purpose of obtaining GC-associated RNA expression patterns, the GEO database was employed. The GSE13861 dataset, the GSE55696 dataset, and the GSE103236 dataset were utilized in subsequent research. The GSE13861 dataset contained 71 GC tumor samples (65 primary gastric adenocarcinoma and 6 gastrointestinal stromal tumors) and 19 adjacent non-tumor samples derived from the GPL6884 platform. The GSE55696 dataset comprised 58 GC cancer samples (19 high-grade intraepithelial neoplasia, 20 low-grade intraepithelial neoplasia, and 19 gastric early-stage carcinoma) and 19 chronic gastritis tissue samples derived from the GPL6480 platform. GSE103236 dataset included 10 GC tumor samples and 9 adjacent non-tumor samples from the GPL4133 platform.

Raw gene mRNA data for 375 GC tissue samples and 32 adjacent non-tumor tissue samples were obtained through the TCGA database (https://portal.gdc.cancer.gov). These samples were taken from patients with a range of stages of the disease.

Due to the fact that the data originated from TCGA and GEO databases, authorization from an ethical committee was not required.

### 2.2. Differentially Expressed Genes (DEGs)

We created one single dataset by combining the previous three, each of which contained 139 GC cancer samples and 47 control samples. We batch standardized the combined datasets using the sva tool in R in order to eliminate any bias that may have existed among the three datasets (version 4.1.3). After that, the limma tool was used to extract DEG information from the GEO and TCGA databases. The criteria for selection were an adjusted *p*-value of 0.05 or lower and a log fold change (FC) of greater than 1. Those DEGs that we are able to meet the threshold were considered to have statistical significance. When attempting to visualize the data, the R software was utilized to generate volcano plots as well as heat maps.

### 2.3. Cell Culture and Transfection

The human gastric cancer cells (MKN-45, AGS, and MGC-803), as well as the normal GES-1 gastric mucosal cells, were generously provided by the American Type Culture Collection (ATCC). These cells were grown in a RPMI-1640 media from Gibco (Grand Island, New York), which included 10% fetal bovine serum (FBS), 100 units per milliliter of penicillin, and 100 milligrams per milliliter of streptomycin (Gibco). All cells were grown at a temperature of 37 degree Celsius in an atmosphere that was 95 percent humidity and 5 percent carbon dioxide. GenePharma (Shanghai, China) developed two shRNAs (sh-HKDC1#1 and sh-HKDC1#2) to target HKDC1, as well as one sh-NC for negative control. These plasmids were transfected into AGS and MKN-45 cells with the use of Lipofectamine 3000 (Invitrogen, Carlsbad, CA).

### 2.4. RNA Extraction and Quantitative RT-PCR (qRT-PCR)

Total RNA was extracted from GC cell lines by using TRIzol (Invitrogen, located in Grand Island, New York), and it was then processed in accordance with the technique that was provided by the manufacturer. cDNA was synthesized by following the instructions given by the manufacturer of the PrimeScript RT Reagent Kit and carrying out the procedure in accordance with those instructions (TaKaRa Bio, Shiga, Japan). In order to carry out real-time PCR, a Roche Light Cycler 480 (Roche) was coupled with an SYBR Green PCR Master Mix. Both of these components were manufactured by Roche (TaKaRa Bio, Shiga, Japan). The calculation was carried out by 2Ct to find the fold change about the mean value. Each experiment yielded three distinct data sets, which were collected separately. The primer sequences are presented down below.

HKDC1:

5′-GCAAGAGACAATCCTGGTACG-3′ (forward).

5′-GTTGCCCTCTGAACGCAATC-3′ (reverse);

Ki67:

5′-ATGCTTGTCGTGTTTTACGGC-3′ (forward).

5′-CGTGGTAATGTAAAGTCCCATGTGTAA-3′ (reverse);

PCNA:

5′-CTGTTAGTAGATGAAACATGGGGG-3′ (forward).

5′-CATCGTGTACGTGCCCAGTGAATGTGTGTG-3′ (reverse);

GAPDH:

5′-GAAAAGATCCCTCCGGAGCAT-3′ (forward).

5′-CTGTGGTGTTTCTCATGCATACG-3′ (reverse);

### 2.5. Cell Count Kit-8 Assay

After the HKDC1 was knocked down, the amount of cell proliferation was measured using the CCK-8 assay, which was created by Dojindo Laboratories in Kumamoto, Japan. We examine the outcomes after just planting four times 10^3^ transfected cells in each well of 96-well plates and leaving them there for 24 hours at a temperature of 37 degrees Celsius with 5 percent carbon dioxide. Following this step, a 10 *μ*l solution of CCK-8 was given to each well, and the cells were cultured for a total of two hours at each of the periods that were stated (0, 24, 48, 72, and 96 h). A spectrophotometer microplate reader (Bio-Rad, California) was utilized for the measurement of cell viability at 450 nm.

### 2.6. Glycolysis Measurement

The levels of glucose uptake, lactate, and ATP generation were each determined in accordance with the instructions that were provided by the respective manufacturers by using the Glucose Uptake Colorimetric Assay Kit (BioVision, Milpitas, CA), the Lactate Assay Kit II (BioVision), and the ATP Colorimetric Assay Kit (BioVision). We used the Seahorse Extracellular Flux Analyzer XF96 (Seahorse Bioscience, North Billerica, MA) to determine the extracellular acidification ratio (ECAR) and oxygen consumption ratio (OCR) of gastric cancer cells. In brief, 2x 10^4^ of the transfected cells were plated into each well of the XF96-well plate and incubated overnight. In order to calculate the ECAR (in mpH/min), glucose, oligomycin (an inhibitor of oxidative phosphorylation), and 2-DG (an inhibitor of glycolysis) were sequentially added to the wells. In order to determine the OCR (in pmol/min), oligomycin, p-trifluoromethoxy carbonyl cyanide phenylhydrazone (FCCP, the reversible inhibitor of oxidative phosphorylation), rotenone (the inhibitor of mitochondrial complex I), and antimycin A (mitochondrial complex III inhibitor) were sequentially added to the wells. The Seahorse XF-96 analysis tool was applied in order to check the results.

### 2.7. Gene Set Enrichment Analysis (GSEA)

In order to evaluate any potential differences, linked pathways, and molecular processes that were discovered in the TCGA cohort, a GSEA was carried out. This was done for the purpose of determining whether or not there were any underlying molecular mechanisms of the glycolysis signature that might be attained. Java SE version 4.1.0 was used to carry out the GSEA, and the following gene sets were included in the analysis: “c5.go.bp.v7.4,” “c5.go.cc.v7.4,” “c5.go.mf.v7.4,” and “c2.cp.kegg.v7.4.” It was determined that a result was statistically significant if it had a |NES| value that was greater than 1 and a *p*-value that was lower than 0.05.

### 2.8. Repeatability Test for Data

The principal component analysis (PCA) is a well-respected statistical method that was used to simplify the data by lowering the degree of interdependence that existed between the variables. In order to determine whether or not the data could be reproduced accurately, PCA was applied.

### 2.9. Statistical Analysis

We analyzed the data with the IBM's SPSS version 21.0 software (Armonk, New York) and presented the findings in the form of the mean and standard deviation. In order to perform comparisons between the groups, we utilized either the Student's *t*-test or a one-way analysis of variance. The value of *p* < 0.05 (2-sided) was used to denote statistical significance.

## 3. Results

### 3.1. Validation of Data

**Figure 1:**
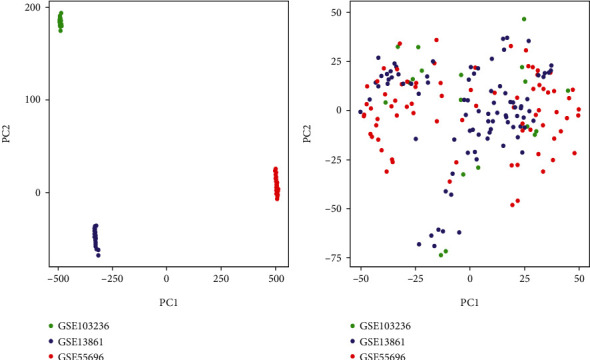
Validation of data. (a, b) PCA showed that the repeatability of the data in GSE103236, GSE13861, and GSE55696 was acceptable.

After using PCA, it was determined that the repeatability of the data in GSE103236, GSE13861, and GSE55696 was adequate (Figure 1(a)). Both the distances between samples in the control group and the distances between samples in the GC group were quite close in the first dimension of the PC analysis.

### 3.2. The Identification of Differentially Expressed Genes from the GEO Database

**Figure 2:**
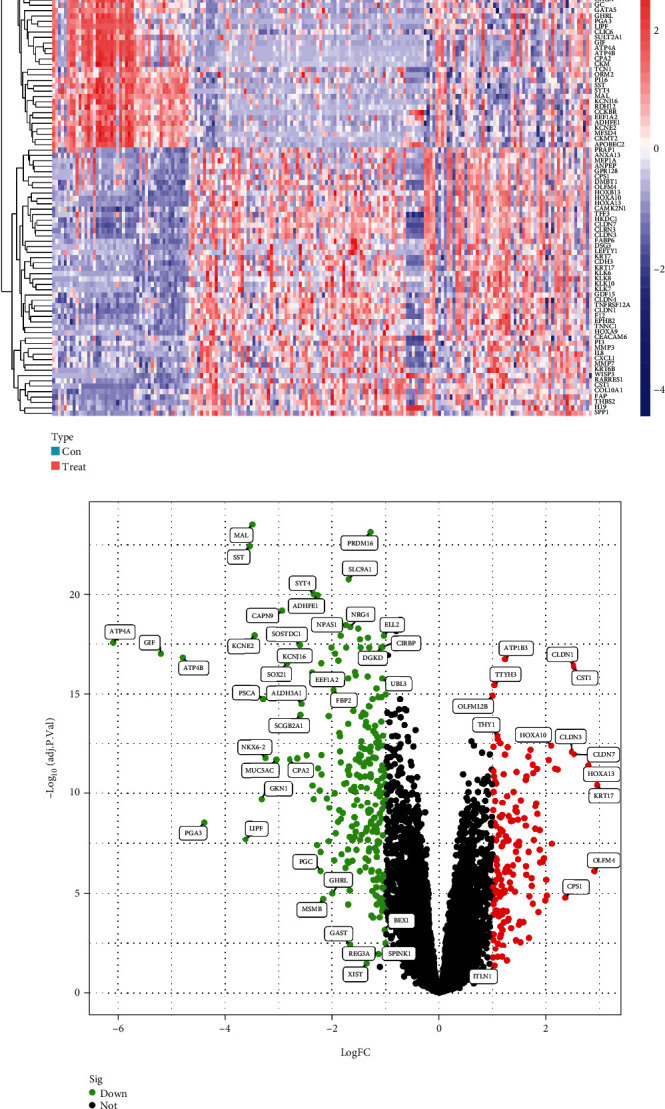
The identification of differentially expressed genes from the GEO database. (a) Heat map presenting DEGs from the merged dataset consisted of GSE103236, GSE13861, and GSE55696. (b) Volcano map presenting DEGs from the merged dataset.

In order to determine which genes are associated with GC using the GEO database, we first combined three datasets, namely, GSE103236, GSE13861, and GSE55696, in order to increase the total number of samples to 139 tumor samples and 47 control samples. After that, the pooled dataset was processed to remove 411 genes that had a *p*-value of less than 0.05 and a logFC of either 1 or −1. (Figure 2(a)). Overall, 253 DEGs saw upregulation, while 158 DEGs experienced downregulation (Figure 2(b); Suppl 1).

### 3.3. Initial Screening of Genes Using Gene Set Enrichment Analysis

**Figure 3:**
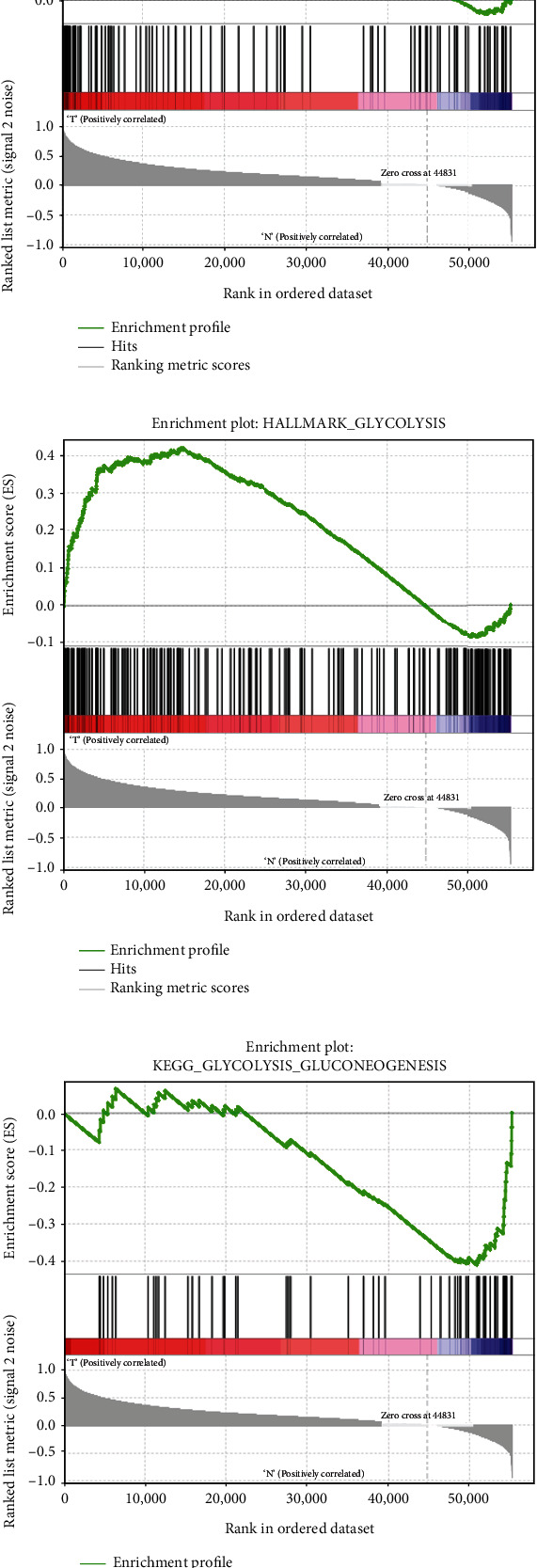
Initial screening of genes using gene set enrichment analysis.Enrichment plots of five gene sets that had significant differences between normal tissues and GC tissues by performing GSEA.

The TCGA provided us with information regarding the levels of expression for 56,753 different mRNAs. Downloading five different glycolysis-related gene sets from MSigDB version 6.2 resulted in a total of 443 genes being found (Suppl 2). With the help of the aforementioned data and GSEA, we were able to establish which gene sets displayed significant differences between GC tissues and the normal tissues that surrounded them. With adjusted *p* values of 0.05, it was shown that four gene sets were significantly enriched from the following pathways: BIOCARTA GLYCOLYSIS PATHWAY, GO GLYCOLYTIC PROCESS, HALLMARK GLYCOLYSIS, KEGG GLYCOLYSIS GLUCONEOGENESIS, and REACTOME_GLYCOLYSIS (Table 1; Figure 3). For the sake of this subsequent research, the 326 genes that belong to these four gene sets were selected.

### 3.4. The Expression Level of Glycolysis-Related Genes in the TCGA Database

**Figure 4:**
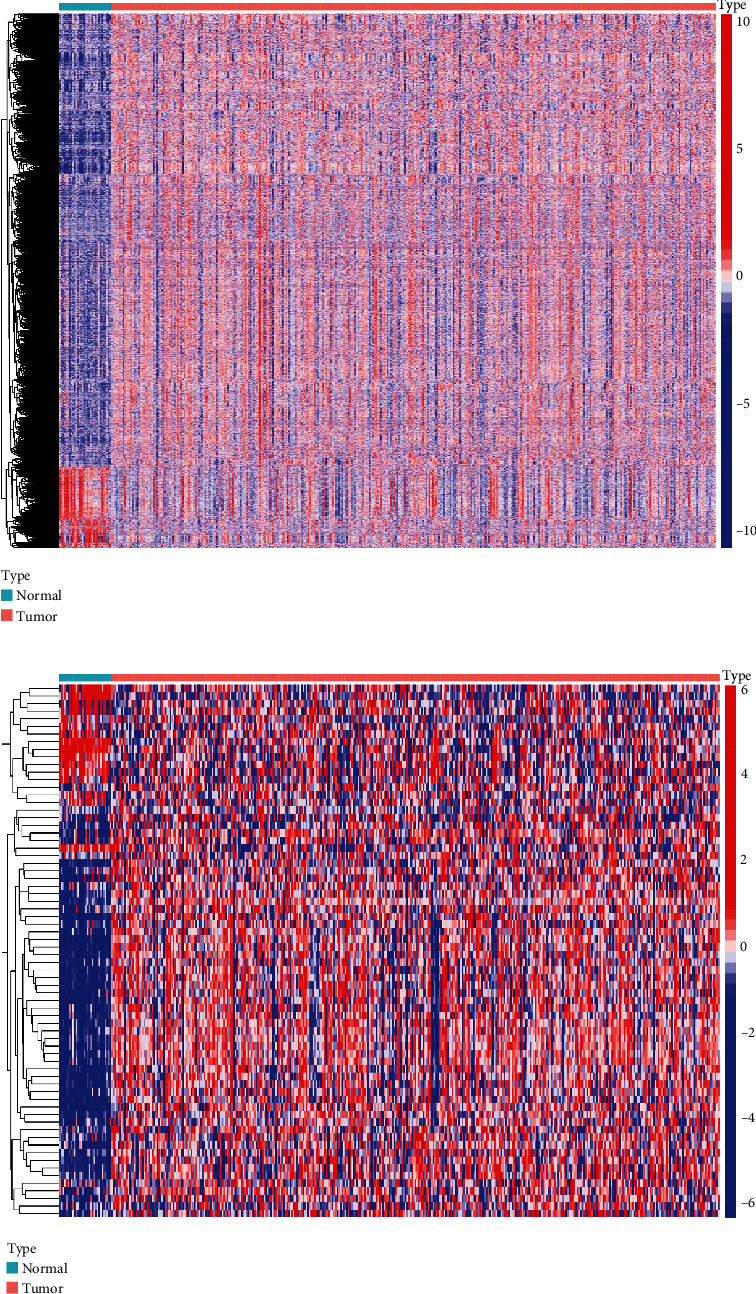
The expression level of glycolysis-related genes in the TCGA database. (a) Heat map of TCGA-derived DEGs with logFC > 1 and *p* − value < 0.05. (b) Heat map of glycolysis-related genes.

Figure 4(a) depicts a heat map of gene expression from the TCGA-STAD dataset. The TCGA database was used to identify the expression levels of 326 glycolysis-related genes (Figure 4(b)).

### 3.5. The Identification of the Hub Gene Associated with Glycolysis

**Figure 5:**
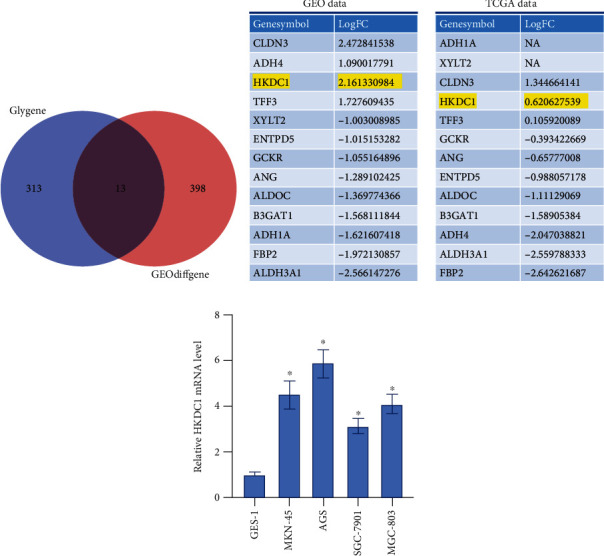
The identification of the hub gene associated with glycolysis. (a) Venn diagram showing 13 DEGs found in both datasets. (b) The expression of glycolysis-related genes in the merged dataset and TCGA database. (c) qRT-PCR analysis of HKDC1 expression in GC cell lines. Data are shown as mean ± SD; ^∗^*p* < 0.05.

The results of a Venn analysis showed that thirteen DEGs taken from the TCGA and GEO datasets were linked to the process of glycolysis (Figure 5(a)). According to the findings of our study, the expression of 13 genes in an integrated dataset and TCGA dataset is shown in Figure 5(b). HKDC1 expression was shown to have significantly increased expression levels in GC tumor tissues from two datasets. Following this, GC cell lines exhibited increased levels of HKDC1 mRNA expression (Figure 5(c)). In addition, HKDC1 expression in MKN-45 and AGS cells was higher than that in GES-1, SGC-7901, and MGC-803 cells. Therefore, MKN-45 and AGS cells were used to carry out the experiment of HKDC1 knockdown.

### 3.6. Downregulation of HKDC1 Inhibits the Growth of Gastric Cancer Cells

**Figure 6:**
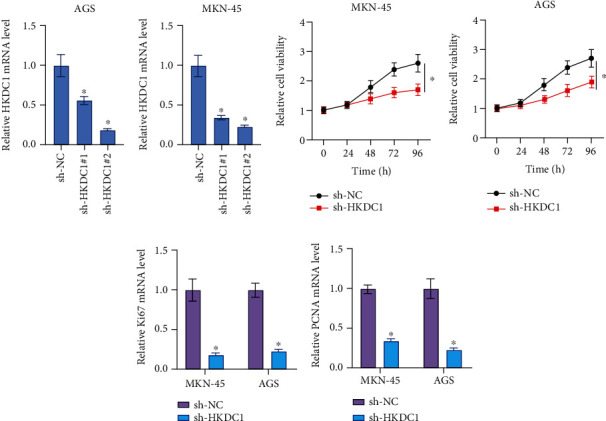
Downregulation of HKDC1 inhibits the growth of gastric cancer cells.(a and b) The qRT-PCR assay was used to evaluate the mRNA level of HKDC1 in AGS and MKN-45 cells. (c and d) CCK-8 assay was used to determine the cell viabilities of AGS and MKN-45 cells. (e and f) The qRT-PCR assay was used to evaluate the mRNA level of Ki67 and PCNA in AGS and MKN-45 cells. Data are shown as mean ± SD; ^∗^*p* < 0.05.

In order to investigate the role that HKDC1 plays in the progression of gastric cancer, AGS and MKN-45 cells were transfected with two different shRNAs directed against HKDC1. Validation of the efficiency of transfection was achieved by the use of a quantitative real-time polymerase chain reaction (qRT-PCR) assay. As can be shown in Figure 6(a), each shRNA was successful in suppressing the expression of HKDC1 (Figure 6(b)). Compared to the sh-NC group, the sh-HKDC1#2 group had lower amounts of mRNA in AGS and MKN-45 cells (Figures 6(a) and 6(b)). Throughout the entirety of this trial, sh-HKDC1#2 was utilized due to the higher knock effectiveness it exhibited. The results of the CCK-8 experiment showed that inhibiting HKDC1 resulted in a considerable and prolonged slowdown in the development of AGS and MKN-45 cells (Figures 6(c) and 6(d)). The expression of proliferation-related proteins, such as Ki67 and PCNA, was consistently decreased after HKDC1 was knocked down (Figures 6(e) and 6(f)).

### 3.7. Downregulation of HKDC1 Inhibits the Glycolysis of Gastric Cancer Cells

**Figure 7:**
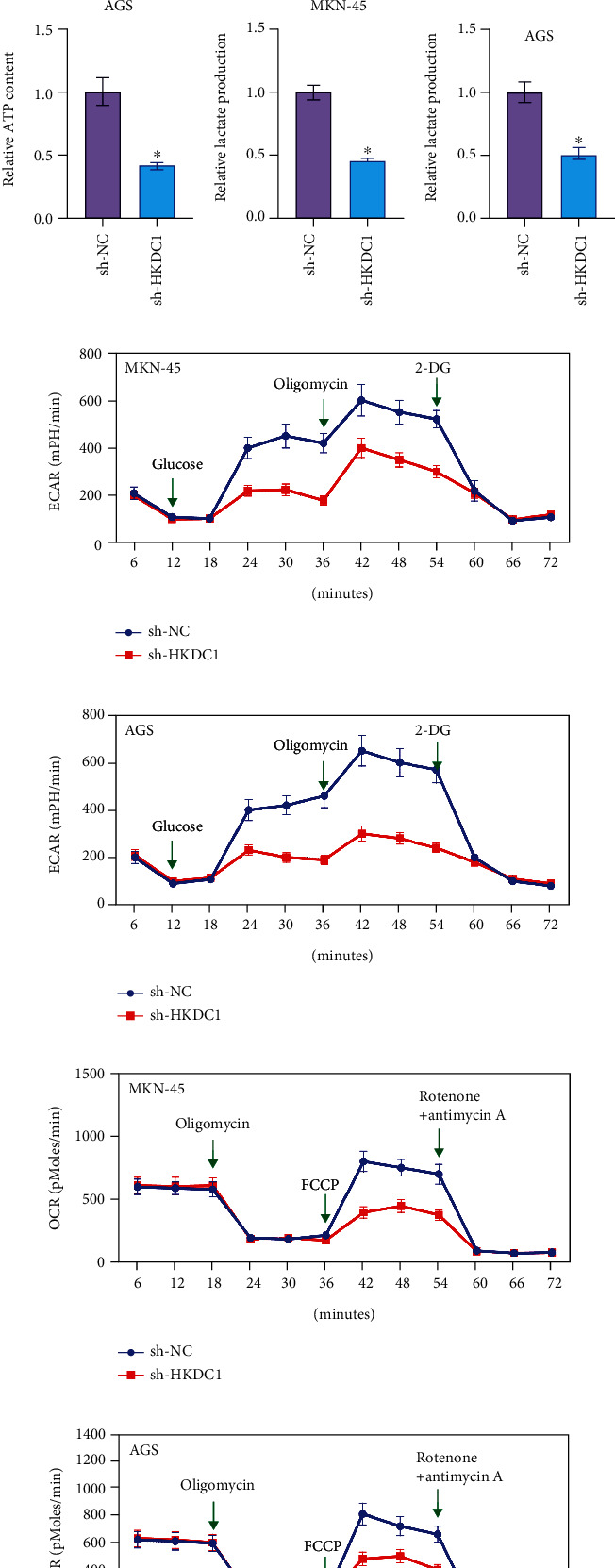
Downregulation of HKDC1 inhibits the glycolysis of gastric cancer cells. (a and b) Glucose uptake of AGS and MKN-45 cells was determined by a glucose uptake colorimetric assay kit. (c and d) ATP synthesis of AGS and MKN-45 cells was determined by ATP colorimetric assay kit. (e and f) Lactate production of AGS and MKN-45 cells was determined by lactate assay kit II. (g and h) The ECAR values of AGS and MKN-45 cells were measured using the Seahorse XF96 analyzer. (i and j) The OCR values of AGS and MKN-45 cells were measured using the Seahorse XF96 analyzer. Data are shown as mean ± SD; ^∗^*p* < 0.05.

The process of glycolysis has been linked to the development of gastric cancer in several studies. As a consequence of our research, we looked into whether or not HKDC1 had any effect on the glycolysis process in gastric cancer cells. The effects of HKDC1 knockdown may be seen in Figures 7(a)–7(f), where it can be seen that glucose absorption, lactate production, and ATP synthesis are all reduced. The electron carrier anion pair (ECAR) and oxygen consumption rate (OCR), which both measure the total flux of glycolysis and mitochondrial oxidative respiration, were also indicators of glycolysis. The results of HKDC1 knockdown are shown in (Figures 7(g)–7(j)), which demonstrate a significant reduction in ECAR and an increase in OCR in AGS and MKN-45 cells, respectively. Our findings indicate that glycolytic activity is hindered in gastric cancer cells when HKDC1 expression is reduced due to downregulation.

## 4. Discussion

More than one million people throughout the world are given a diagnosis of gastric cancer each year, making it a major public health concern [5]. In spite of the fact that both the incidence rate and the mortality rate of gastric cancer have been steadily declining over the course of the past five years, it continues to be the most common and deadly tumor in the world [15]. As a consequence of this, it is of the utmost importance to get a deeper comprehension of the molecular processes that underlie the development of gastric cancer, as well as to locate innovative clinical screening and diagnostic targets.

The study of glycolysis using bioinformatics led to the discovery of 13 genes related to the process. There was a discernible uptick in the levels of expression for CLDN3, ADH4, HKDC1, and TFF3. The genes CLDN3 [16], ADH4 [17], and TFF3 [18] have all been implicated in the development of gastric cancer. Next, HKDC1 expression was significantly increased in the GEO and TCGA databases. Our investigation into the role of HKDC1 in gastric cancer was highly exciting because no one had ever reported on its significance before.

In addition, the oncogenic effect of HKDC1 in hepatocellular [19] and colorectal cancers [20] served as a driving force behind our decision to investigate the functional importance of HKDC1 in gastric cancer. During this examination, we found that the gene HKDC1 was highly expressed in the tissues and cell lines of GC, although its expression was relatively low in the normal tissues that were located in the vicinity. These preliminary data suggest that the gene HKDC1 plays an oncogenic role in gastric cancer. Furthermore, due to its distinctive properties, HKDC1 is ideally suited to serve as a therapeutic target for gastric cancer. It is generally accepted that metabolic reprogramming, such as glycolysis in aerobic circumstances, is an indication of tumor growth in a wide variety of cancers. This is because glycolysis is an aerobic process. The increased glycolysis that occurs in cancer cells leads to increased glucose intake and lactate formation [7]. This alters the metabolic requirements of the cancer cells, which in turn leads to increased invasion and metastasis. According to the findings of a recent analysis, HKDC1 is responsible for catalyzing glucose phosphorylation and the metabolism of cellular energy, both of which are critical in the development and spread of cancer [12]. According to the findings of the current research, HKDC1 is associated with both the process of proliferation and glycolysis in gastric cancer. As a result, HKDC1 may serve as a potential research target for the detection and treatment of gastric cancer, an area that calls for additional investigation.

There is mounting evidence to support the hypothesis that aerobic glycolysis plays a key part in the carcinogenic process, most notably in the development of gastric cancer. Glycolysis reprogramming is predominately glycolytic even when there is a significant amount of oxygen present in cancer cells [21]. It has been proven that oncogenic drive is the primary cause of the cleavage of aerobic glycolysis. This increases cancer cell proliferation and survival by delivering more intermediates for particular biosynthetic pathways and adaptability to hypoxic settings [22, 23]. Cancer cells have a distinctive metabolic phenotype that allows them to maintain the malignant biological processes for that they are responsible. This phenotype includes increased glucose uptake, lactate release, ATP production, and ECAR [4]. We demonstrate in this study that removing HKDC1 has an effect on the proliferation of cells in vitro. In addition, the downregulation of HKDC1 in gastric cancer cells resulted in a significant reduction in glucose uptake, lactate production, ATP synthesis, and ECAR, as well as an increase in OCR and modest inhibition of glycolysis. The initial indication that HKDC1 is a tumorigenic gene in gastric cancer comes from these studies.

## 5. Conclusion

Taken as a whole, the inhibition of cell proliferation and glycolysis brought about by the downregulation of HKDC1 provided a potential new avenue for the investigation of gastric cancer as a research target.

## Figures and Tables

**Table 1:** Gene sets enriched in GC.

GS follow link to MSigDB	SIZE	ES	NES	NOM *p*-val	FDR *q*-val
BIOCARTA_GLYCOLYSIS_PATHWAY	3	0.35	0.58	0.9409	0.9409
GO_GLYCOLYTIC_PROCESS	106	0.57	1.91	0.0056	0.0056
HALLMARK_GLYCOLYSIS	200	0.42	1.36	0.1439	0.1439
KEGG_GLYCOLYSIS_GLUCONEOGENESIS	62	-0.41	-1.28	0.1821	0.1821
REACTOME_GLYCOLYSIS	72	0.68	1.97	0.0040	0.0040

## Data Availability

The data used to support the findings of this study are available from the corresponding authors upon request.

## Supplementary Materials

Supplementary Materials Suppl 1: list of significantly changed DEGs from the GEO database (*p* < 0.05). Suppl 2. List of significantly changed DEGs after screened using Gene Set Enrichment Analysis (*p* < 0.05).
